# Radiological staging in breast cancer: which asymptomatic patients to image and how

**DOI:** 10.1038/sj.bjc.6605323

**Published:** 2009-09-29

**Authors:** T Barrett, D J Bowden, D C Greenberg, C H Brown, G C Wishart, P D Britton

**Affiliations:** 1 Department of Radiology, Box 219, Addenbrooke's Hospital, Cambridge University Teaching Hospitals NHS Foundation Trust, Hills Road, Cambridge CB2 0QQ, UK; 2 Eastern Cancer Registration and Information Centre, Shelford Bottom, Cambridge CB22 3AD, UK; 3 Cambridge Breast Unit, Box 97, Cambridge University Hospitals NHS Foundation Trust, Hills Road, Cambridge CB2 0QQ, UK; 4 Cambridge Biomedical Research Centre, National Institute of Health Research, Hills Road, Cambridge CB2 0QQ, UK

**Keywords:** radiological staging, asymptomatic, breast cancer, CT

## Abstract

**Background:**

Approximately 4% of patients diagnosed with early breast cancer have occult metastases at presentation. Current national and international guidelines lack consensus on whom to image and how.

**Methods:**

We assessed practice in baseline radiological staging against local guidelines for asymptomatic newly diagnosed breast cancer patients presenting to the Cambridge Breast Unit over a 9-year period.

**Results:**

A total of 2612 patients were eligible for analysis; 91.7% were appropriately investigated. However in the subset of lymph node negative stage II patients, only 269 out of 354 (76.0%) investigations were appropriate. No patients with stage 0 or I disease had metastases; only two patients (0.3%) with stage II and ⩽3 positive lymph nodes had metastases. Conversely, 2.2, 2.6 and 3.8% of these groups had false-positive results. The incidence of occult metastases increased by stage, being present in 6, 13.9 and 57% of patients with stage II (⩾4 positive lymph nodes), III and IV disease, respectively.

**Conclusion:**

These results prompted us to propose new local guidelines for staging asymptomatic breast cancer patients: only clinical stage III or IV patients require baseline investigation. The high specificity and convenience of computed tomography (chest, abdomen and pelvis) led us to recommend this as the investigation of choice in breast cancer patients requiring radiological staging.

Breast cancer is second only to lung cancer in worldwide prevalence. It represents 16% of all UK cancers and is the most common cancer in women, accounting for 31% of all cancers (WHO, 2003, 2006). The incidence of breast cancer amongst women in the UK was 45 660 in 2005 (ONS, 2008). Approximately 4% of breast cancer patients will have detectable metastatic disease at the time of diagnosis; the majority of these patients will have signs or symptoms of metastases (Ravaioli *et al*, 2002). The most common first sites of distant metastases are bone, lung, brain and liver; up to 10% of patients with metastatic disease will have lesions at multiple sites (Patanaphan *et al*, 1988). Conventionally, high-risk patients have been screened for occult metastases in these organs by using chest radiographs (CXR), liver ultrasound (US) and bone scintigraphy (BS) (Puglisi *et al*, 2007). More recently, techniques such as computed tomography (CT), magnetic resonance imaging and ^18^FDG-positron emission tomography (Avril *et al*, 2007) have started to be used in certain circumstances, or for problem-solving.

The likelihood of detecting metastatic disease at the time of initial diagnosis is highly dependant upon the extent of loco-regional disease and the presence of symptoms. The detection of such metastases has a profound effect upon patient management and prognosis, however, there is little evidence to guide decisions on which patients should be staged. Where symptoms are suggestive of metastatic disease, appropriate investigations targeted to the respective organs should be instigated. However, the vast majority of newly diagnosed patients do not exhibit symptoms or signs of metastatic disease. As metastatic rates overall are so low, staging all patients would result in unacceptable expenditure, increased workload, patient anxiety and radiation burden. Imaging staging of patients needs to be targeted towards those most likely to have disseminated disease. The aim of this paper was to assess practice in baseline radiological staging against local guidelines of a large cohort of breast cancer patients stratified by clinico-pathological stage and additionally determine the likelihood of detecting metastases and, as importantly, obtaining a false-positive result. We also aimed to explore the current trend to use CT rather than conventional assessment with CXR, US liver and BS.

## Materials and methods

A list of all female patients diagnosed with breast cancer during a 9-year period, between 1 January 1999 and 31 December 2007, was obtained from the Eastern Cancer Registration and Information Centre (ECRIC) database. Each patient was prospectively allocated a clinico-pathological staging classification by the ECRIC medical director and clinical oncologist (author CHB). The classification was undertaken 6 months following initial diagnosis by reviewing the medical records and was based on clinical, imaging and pathological data. The overall distribution of patients between stages is comparable to data published by the National Cancer Data Base, USA (Table 1).

Staging was allocated according to the TNM (tumour, node, metastasis) staging system (0–IV) published in the American Joint Committee on Cancer (AJCC) staging manual. The AJCC sixth edition was published in 2002 (Greene *et al*, 2002), replacing the fifth edition from 1997 (Fleming *et al*, 1997) and contained some changes particularly relevant to our study. In the sixth edition ‘N stages’ were changed to give greater emphasis to the number of nodes involved: 4–9 nodes (or any internal mammary lymph nodes) became N2 disease, and ⩾10 axillary nodes now qualified as N3 disease. N1 disease classifies the patient as stage II, whereas N2 or N3 disease places a patient in the stage III category (Supplementary Table 1). For example, patients with four positive axillary lymph nodes would be stage IIb using the criteria of the fifth edition, but stage IIIa under the sixth edition. To avoid confusion, we retained the criteria used in the fifth edition, but subdivided our results for stage II disease into patients with three nodes or less (stage II-i) or more than four nodes (stage II-ii) involved. It should be noted that patients with N1 status can still have stage III or IV disease, if the tumour is >5 cm diameter, or involves the skin or chest wall.

Patients in whom an accurate stage could not be defined due to insufficient information and patients with symptoms, signs or abnormal blood work indicative of metastatic disease were excluded from the data analysis. The Cambridge Breast Unit is a tertiary referral centre and treats some patients initially diagnosed with breast cancer at another centre. As an accurate investigation history could not always be reliably obtained, patients that did not receive their primary diagnosis of breast cancer at this institution were also excluded. The final exclusion criteria included patients whose breast cancer was diagnosed on the basis of co-incidental abnormal imaging results that suggested metastases and the search for a primary cancer ensued.

With clinical stage being assigned retrospectively, 6 months after diagnosis, patients where imaging had shown metastases were classified as stage IV, however these patients may have initially presented with a lower stage of disease clinically. Thus all stage IV cases were reviewed on an individual basis and patients were re-assigned to the relevant lower stage group based on the information that would have been available at presentation. It should be noted that patients can still present with clinical stage IV disease, for example inflammatory breast cancer, thus seven patients remained in this group following the application of exclusion criteria and stage re-assignment.

**Figure 1:**
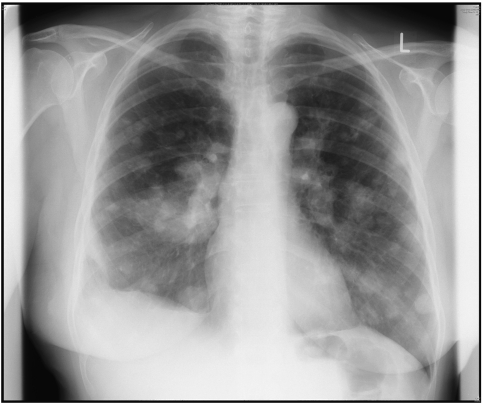
An ‘unequivocal’ true positive. Chest X-ray showing multiple metastases throughout both lungs in a patient with stage IV breast cancer.

**Figure 2:**
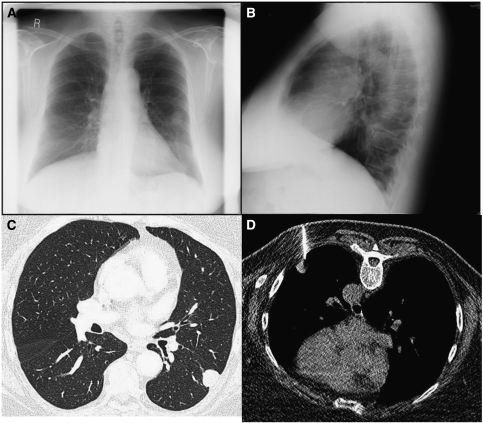
True positive. Chest X-ray reveals a ‘coin’ lesion in the left mid-zone (**A**), also demonstrated on the lateral radiograph (**B**). CT examination (**C**) on lung-window settings supports the diagnosis of a likely lung metastasis, later confirmed by CT-guided biopsy, performed with the patient lying prone (**D**).

**Figure 3:**
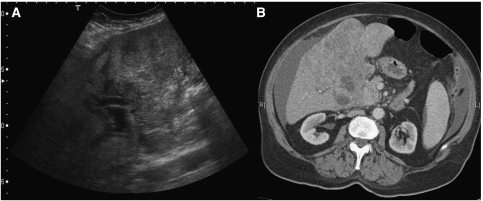
True positive. Stage IV breast cancer patient. Ultrasound liver shows irregular lesions within the liver (**A**), subsequent CT (**B**) confirms liver metastases, ascites also demonstrated.

**Figure 4:**
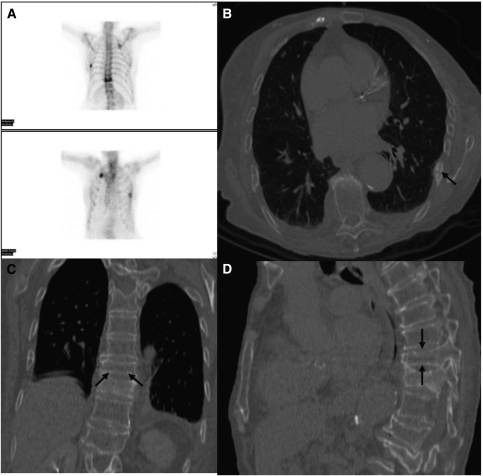
False positive. Bone scintigram (**A**) and CT chest examination (**B**, **C**, **D**) in a patient with stage IV breast cancer. The bone scan was reported as showing hot spots in the T10 vertebrae, left posterior seventh and right anterior second ribs, consistent with metastases. The follow-up CT scan revealed these changes to be due to rib fractures (**B**), and degenerative disease (**C**, **D**) only.

Baseline staging investigations were arbitrarily chosen to be those taking place within 3 months of the date of breast cancer diagnosis. This was considered to be a reasonable time period for management to be discussed in multi-disciplinary meetings, surgery to take place, and histopathological results to be available and subsequent imaging tests to be performed. Patients were analysed on a case-by-case basis, with imaging investigations, indications and results logged. A true-positive result was determined as being one that unequivocally confirmed metastatic disease on the basis of the respective imaging investigation (Figure 1), or one that was proven by subsequent imaging or histology (Figures 2 and 3). A false-positive result was taken as being any staging investigation that was initially reported as being either abnormal or indeterminate, but upon subsequent investigation(s) was proven to be negative (Figure 4).

The local staging guidelines at the Cambridge Breast Unit (updated in 1999) used as the ‘gold standard’ for our assessment are as follows:

- Pre-operative CXR is not necessary as a staging investigation unless past medical history warrants, or symptoms warrant or there is a high risk of an ITU admission (reference *Making the Best Use of a Department of Clinical Radiology*, Royal College of Radiologists)
- Patients with T4 tumours or malignant lymphadenopathy should be considered for pre-operative staging with CXR, liver US and bone scan
- Patients with more locally advanced breast cancer should have investigations directed at findings underlying asymptomatic metastases where this may influence management

Thus, patients with positive lymph nodes or T4 tumours (involvement of chest wall or skin) are eligible for baseline staging investigations according to local guidelines. This includes all tumours that are N1–3 and/or T4 and any request for CXR, bone scan, US or CT in such patients was considered appropriate. However, staging was not deemed appropriate in stage 0, I and stage II lymph node negative patients; a CXR for pre-operative assessment was considered appropriate if indicated under the RCR guidelines (RCR, 2007).

Finally we estimated the health-care costs of detecting metastases by stage of disease and mode of imaging staging. This was based on local costing taking into consideration staffing, consumable and hardware expenses. From the true-positive rates achieved in this study we have calculated how much would need to be spent to detect a single patient with metastatic disease for each disease stage. These estimates have been made for staging by both standard (CXR £80, US liver £176 and BS £184) methods and for CT alone (chest, abdomen, pelvis £271). We have also included in all these estimates the cost of additional imaging generated by the false-positive results in this study.

## Results

Of 3398 patients with newly diagnosed breast cancer over the 9-year study period, 2612 cases were eligible for data analysis (Table 1). Appropriate investigations undertaken in stages 0, I, and II-i (stage II with ⩽3 lymph nodes), that is patients who should receive only a CXR (if clinically indicated) and no additional staging investigations according to local guidelines, were as follows: 325 out of 348 (93.4%) for stage 0, 881 out of 992 for stage I (88.8%) and 779 out of 859 for stage II-i (90.7%). For the subgroup of stage II-i with no lymph nodes involved only 269 out of 354 requests (76.0%) were appropriate. Overall for stages 0, I and II-i, 1985 out of 2199 (90.3%) patients were appropriately investigated. Including the 413 patients with stages II-ii, III and IV disease, who were all appropriately investigated, the overall compliance with local guidelines for baseline screening in asymptomatic breast cancer patients was 2398 out of 2612 (91.8%).

Of the 1340 patients with stages 0 and I (674 of whom had a least one investigation) there were no true-positive results but 17 patients had false-positive results. Of the 859 patients with stage II-i disease there were only 2 potential true-positive results (0.3% of cases). The majority (607) of these patients had at least one investigation, and there were 23 (2.7%) false-positive results in this group. There were 10 (6%) true-positive results and 22 (13.1%) false-positive results in patients with stage II-ii; 26 (13.9%) true positives and 24 (12.8%) false positives in stage III and 4 (57%) true positives, with no false-positive results in patients with stage IV disease (Table 2).

**Figure 5:**
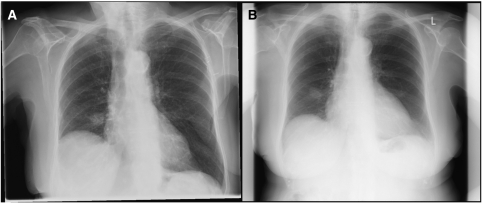
One of two patients with stage II-i disease with a ‘true-positive’ diagnosis. The soft tissue lesion in the right lower zone was reported as being a metastatic lesion on baseline CXR shortly after the patient presented with primary breast cancer in 2001 (**A**). However, in 2007 the lesion had not significantly changed in size (**B**) and was retrospectively described as being ‘unlikely to be malignant’.

The two potential ‘true’-positive results in stage II-i patients require further explanation. The first patient was 87 years old at diagnosis and presented with a 22 mm, node negative, grade 2 tumour. A soft tissue lesion was seen in the right lower zone on baseline CXR and reported as representing a metastasis and therefore classified as a true positive in this study. However, 6 years after initial presentation, a repeat CXR showed that the lesion had not changed in size thus casting doubt over the initial malignant diagnosis (Figure 5). In the interest of consistency we have retained the initial classification for our analysis, however it is possible that this represents a false- rather than true-positive case. The second true-positive patient in the stage II-i group was 62 years old at diagnosis, and presented with a 38 mm, node negative, grade 3 tumour. Chest radiographs suggested lung metastases, subsequently confirmed at CT, which additionally showed peritoneal metastases. The patient was a smoker with a history of hypertension, thus met local guidelines for a pre-operative CXR. A subsequent clinic letter documented erythema and thickening of the axilla; skin involvement would make the tumour T4 (stage IIIb), thus it is possible that this case represents an error in stage assignment.

There was a difference in the accuracy of the four different imaging modalities used. There were 3 true-positive (0.2%) and 20 (1.3%) false-positive results out of 1556 CXRs, 6 true-positive (1.8%) and 13 (3.8%) false-positive results out of 339 liver US, 23 true-positive (6.2%) and 51 (13.7%) false-positive results out of 373 bone scans, and 21 true-positive (26.9%) and 3 (3.8%) false-positive results out of 78 CTs (Table 3). Computed tomography was the only modality in which the percentage of true-positive results was higher than the false-positive results. Computed tomography also had a lower rate of false-positive results (3.8%) than US and BS. Of the three false-positive CT results two described small pulmonary nodules of indeterminate significance, which were unchanged on follow-up CT, and one described a liver lesion that was too small to characterise, but was shown to be a cyst on subsequent US. There was a noticeable trend during the study period to request more CT scans, with a corresponding reduction in requests for CXR, US and bone scans.

In this review there were 23 patients with true-positive bone scan results; of these every case had at least one metastasis in the field that would be covered by CT of the chest/abdomen/pelvis. Retrospective analysis of all bone scan results (including some symptomatic patients who were later excluded) revealed that of 411 scans performed, 257 were normal, 41 showed degenerative change, 52 were true-positive results and 61 were false-positive results. In the 52 cases where the bone scan was found to be a true positive, all patients had at least one metastasis within the field that would be covered by CT of the chest, abdomen and pelvis.

The estimated health-care cost of detecting metastases by stage of disease and mode of imaging staging is shown in Table 4. Calculations were carried out on the true-positive rates and the added expense generated by false-positive imaging results. The CXR, BS and liver US false-positive rates in this study were 1.3, 13.7 and 3.8% respectively. Every false-positive CXR, BS and US cost £89, £132 and £190, respectively, in additional imaging investigations. The CT false-positive rate was 3.8% and every false-positive CT costs £203 in additional imaging investigations. As no metastatic disease was detected in any patient with stage 0 or I disease the theoretical cost of detecting a metastasis was infinity. The cost of detecting one true-positive in patients with stage II-i disease (£200 393 by standard staging) is substantially higher than for stage II-ii patients (£8492); thereafter, the cost of detecting a metastasis continues to fall with increasing stage. It can also be seen that there is an overall cost saving if CT is used to replace the standard combination of CXR, BS and US.

## Discussion

We assessed local practice for the radiological staging of women presenting with a new diagnosis of breast cancer, without symptoms of metastases, over a 9-year period. Overall, the yield for detecting metastases is low in such asymptomatic patients, with no occult metastases detected in any patient with stage 0 or I disease. Furthermore, only two patients with stage II-i disease had possible evidence of occult metastases. In fact, in patients with stage 0 to II-i such screening is likely to lead to a greater degree of false-positive results: 40 (out of the 2199 patients). The yield is improved in those presenting with a higher stage of disease: 40 out of 413 patients with stage II-ii, III, or IV disease had true-positive results (*vs* 46 false positives). These results show the benefit of a risk-stratified staging protocol for early breast cancer but underline the importance of making inclusion criteria clear and less open to interpretation. In this way the majority of occult metastases can be detected with minimal false positives, incidental findings and unnecessary radiation exposure.

The local guidelines for our review were, in retrospect, non-specific and open to interpretation. Although the inclusion of patients with T4 disease or any evidence of malignant lymphadenopathy is very clear, the inclusion of ‘patients with more locally advanced disease’ is open to interpretation. In fact both national and international guidelines on the baseline imaging of breast cancer tend to be either open to interpretation or lack consensus. NICE guidelines from 2002 recommend that patients with T4 tumours (stage III+) should have staging investigations, their 2009 update additionally accepts that there is currently insufficient evidence to support the choice of one imaging modality over another (NICE, 2002, 2009). The Scottish Intercollegiate Guidelines Network also states that only stage III+ may require staging (SIGN, 2005). The National Comprehensive Cancer Network guidelines suggest that staging investigations are not required in asymptomatic stage I disease, nor many other patients with early stage breast cancer, and should only be considered in those with symptoms or T3, N1, M0 disease (stage IIIa) (NCCN, 2009). Conversely, the British Association of Surgical Oncology states that no asymptomatic patients should be staged, aside from pre-operative CXR (BASO, 2005).

These varying recommendations for imaging staging for occult metastases in women with newly diagnosed breast cancer reflect the paucity of evidence on this issue in the setting of early stage disease without symptoms suggestive of metastases. Internationally, the most robust guidelines seem to be those proposed by the Cancer Care Ontario Practice Guidelines Initiative (CCOPGI) (Myers *et al*, 2006). These guidelines are also endorsed by the National Guideline Clearinghouse – an initiative of the Agency for Healthcare Research and Quality which is affiliated with the American Medical Association and America's Health Insurance Plans. The guidelines are based on a literature search of peer-reviewed articles on the baseline investigation of asymptomatic breast cancer patients up to 2003. They recommend no investigations for stage 0 and I patients, a BS for stage II disease and CXR, US and BS for stage III tumours.

Having decided which patients to investigate, it is then important to consider which modality or modalities are most appropriate for radiological staging. The trend we have observed locally is to move away from the more traditional methods of investigation with CXR, US and BS, to use CT of the chest, abdomen and pelvis. Certainly the CT option is logistically easier for the patient – it involves one visit to the department and the images can be acquired in a matter of seconds. If patients are required to have a CXR, US and BS, this will often involve separate attendances (often on separate days) to different areas of the hospital and, in the case of bone scan, returning 4 h after initial injection of isotope to complete the study. Indeed, a study into patient preference comparing CT with BS revealed that 75% patients preferred their experience of CT, compared with only 5% at BS (20% no preference) (Groves *et al*, 2006). There is no question that CT chest is more sensitive and specific than CXR for diagnosing lung metastases, nevertheless, it does provide an increased radiation burden. Overall CT probably has a higher sensitivity than US for liver lesion detection. However, US will retain a role in targeting the biopsy of indeterminate lesions, or evaluation of lesions too small to characterise by CT.

Perhaps more controversial is the use of CT instead of BS. Computed tomography advances enable improved detection of bone metastases and there is evidence that this is equivalent to BS, with some authors concluding that the latter may be omitted (Groves *et al*, 2006; Bristow *et al*, 2008). Bone scintigraphy covers the entire skeleton (including long bones, neck and skull), however, of the 52 true-positive bone scan results in our study all had at least one metastasis within a field that would be covered by CT chest/abdomen/pelvis. Furthermore, detection of bone metastases at an asymptomatic stage has not been shown to prolong survival (Sato *et al*, 2003). Another issue is the increased radiation dose of CT. The effective dose of CT of the chest, abdomen and pelvis is 9.9 mSv (Shrimpton *et al*, 2006), in comparison with a bone scan (740 MBq injected) at 4.2 mSv (ICRP, 1988), and CXR at 0.02 mSv (HPA, 2005). The long-term effects of radiation exposure are a concern; however, this is likely to be less relevant in patients with higher-stage breast cancer.

Out of 411 bone scans, 298 were negative, 52 were true positives and 61 were false positives. These results are similar to findings of Puglisi *et al* (2005) where 26 true-positive and 25 false-positive results were reported in 412 breast cancer patients of all stages. The false-positive rate of bone scans (13.7%) is much higher than that of CT (3.8%) in our study. The increased specificity of CT may reduce follow-up investigations, which could partially offset the increased radiation dose, limit the psychological burden of false-positive results and reduce the need for further invasive testing. A meta-analysis of bone scan results in asymptomatic breast cancer patients by the CCOPGI group revealed differences in studies performed before 1980, with high pick-up rates, and those after, with a low rate of metastatic detection (Myers *et al*, 2006). The differences may be explained by differing thresholds for reporting positive results. In current practice, any equivocal bone scan result will lead to further imaging and possibly biopsy for confirmation of malignancy; thus in the earlier studies it is possible that some positive results were in reality false-positive results. Also the advent of screening programmes is expected to alter the demographics of the staging groups, with a higher proportion of patients presenting with earlier stage disease. Similar factors may help explain the relatively small incidence of metastases observed in our patient population, with low-stage disease: 0% of patients in stages 0 and I and only 0.3% of patients in stage II shown to be true positives.

This study was not designed for direct comparison of test accuracy, however, the apparent advantages of CT in relation to improved sensitivity and patient convenience, along with the local trend towards this form of imaging, have led us to recommend this as the baseline imaging modality of choice in patients presenting to our unit with asymptomatic newly diagnosed breast cancer. Given the relative lack of evidence directly comparing CT and BS for bone metastases in breast cancer, we are extending the CT protocol in breast cancer patients to include the supraclavicular fossa and proximal femur. The cost estimates in Table 4 are included to give an indication of the relative costs of implementing different imaging strategies based upon disease stage. Regardless of which stage of disease is used as a cut-off for imaging, our data suggest that implementing a strategy of using CT rather than conventional staging investigations (CXR, BS and US) would result in health-care cost saving.

## Conclusion

The result of this study together with published evidence and existing guidelines has led us to adopt the following policy for the imaging of asymptomatic patients with newly diagnosed breast cancer, which we hope will maximise metastatic detection while minimising harmful side effects:

- Pre-operative CXR is not necessary as a staging investigation – follow RCR guidelines (RCR, 2007)
- Patients with clinical stages III and IV disease (Greene *et al*, 2002) should undergo staging with CT of the chest (to include the supraclavicular fossa), abdomen and pelvis (including proximal femur). This includes patients with tumours that are:
  - Larger than 5 cm and involve at least one axillary lymph node
  - Any size and involve ⩾4 axillary lymph nodes
  - Any size and involve at least one internal mammary, infraclavicular or supraclavicular lymph node
  - Any size and involve the skin or chest wall (includes inflammatory breast cancer)
  - In patients with stage III or IV disease where the results will not affect management, staging investigations are not required

## Figures and Tables

**Table 1:** Assessed patient numbers

**Stage**	**Total cases 1999–2007**	**Total (%)**	**Patients meeting inclusion criteria**	**Total (%)**	**NCDB case data** a	**NCDB % data** a
0	432	12.7	348	13.3	57 796	17.1
I	1155	34	992	38	140 122	41.4
II	1388	40.8	1041	39.8	106 116	31.3
III	244	7.2	224	8.6	22 758	6.7
IV	145	4.3	7	0.3	11 780	3.5
Unknown	34	1	0	0	0	0
Overall	3398	100	2612	100	338 572	100

Abbreviation: NCDB=National Cancer Data Base.

Inclusion criteria: female patients with a new diagnosis of breast cancer established between 1 January 1999 and 31 December 2007. Exclusion criteria: diagnosis not established at Addenbrooke's, or accurate stage could not be established – see text for full explanation.

Reports, v1.1. Chicago, IL, 2002. http://www.facs.org/cancer/ncdb/publicaccess.html.

a Comparative figures taken from the National Cancer Data Base, USA 1998–1999: cases diagnosed in 1247 US nationwide hospitals in 1998 and 1999: Commission on Cancer, American College of Surgeons. NCDB Benchmark.

**Table 2:** Number of investigations performed by modality and stage of disease and true- and false-positive results by stage of disease

**Stage**	**0**	**I**	**II-i**	**II-ii**	**III**	**IV**	**Total**
Total no. of patients	348	992	859	182	224	7	2612
							
*Staging investigations*							
CXR	136	527	580	148	160	5	1556
US	0	19	101	100	114	5	339
BS	0	22	109	113	125	4	373
CT	3	6	19	15	34	1	78
							
*Total no. of patients having staging investigations*							
True +ve (%)	0 (0)	0 (0)	2 (0.3)	10 (6)	26(13.9)	4 (57)	
False +ve (%)	3 (2.2)	14 (2.6)	23 (3.8)	22 (13.1)	24 (12.8)	0 (0)	

Abbreviations: BS=bone scintigraphy; CT=computed tomography; CXR=chest radiograph; US=ultrasound.

Stage II-i=patients with stage II disease by AJCC sixth edition (Greene *et al*, 2002): ⩽3 lymph nodes positive, stage II-ii=patients with stage II disease by AJCC fifth edition (Fleming *et al*, 1997), but stage III disease by AJCC sixth edition, i.e. ⩾4 positive lymph nodes.

**Table 3:** True- and false-positive results by modality (includes all stages of disease)

	**CXR**	**US**	**BS**	**CT**
Total	1556	339	373	78
True +ve (%)	3 (0.2)	6 (1.8)	23 (6.2)	21 (26.9)
False +ve (%)	20 (1.3)	13 (3.8)	51 (13.7)	3 (3.8)

Abbreviations: BS=bone scintigraphy; CT=computed tomography; CXR=chest radiograph; US=ultrasound.

**Table 4:** Estimate of health-care costs to detect metastases by stage of disease and by mode of imaging

	**TNM stage**					
	**0**	**I**	**II-i**	**II-ii**	**III**	**IV**
True-positive rate for detection of metastases (%)	0	0	0.2	5.5	11.6	57.0
						
*Estimated cost of detecting 1 patient with metastatic disease by*						
‘Standard’ staging Investigations (CXR, US, BS)	Infinite	Infinite	£200 393	£8492	£4021	£817
CT staging alone	Infinite	Infinite	£119 744	£5074	£2405	£488

Abbreviations: BS=bone scintigraphy; CT=computed tomography; CXR=chest radiograph; TNM=tumour, node, metastasis; US=ultrasound.

Estimates for detecting a single patient with metastatic disease for each disease stage based on local costings and the respective true- and false-positive rates by modality (see text for costing estimates).
