# Supplementary material for: Radiological staging in breast cancer: which asymptomatic patients to image and how
Source: Br J Cancer. 2009 Sep 29;101(9):1522–8. doi: 10.1038/sj.bjc.6605323 (PMC2778507; doi:10.1038/sj.bjc.6605323)
Supplement: Supplementary Table 1 [file 6605323x1.doc]

Tables

| **Stage** | **TNM** | **Tumour descriptions** |
| --- | --- | --- |
| **0** | T-Tis, N0, M0 | DCIS or Paget’s disease of the nipple (with no underlying tumour mass) |
| **I** | T1, N0, M0 | Tumour <2cm |
| **IIA** | T0, N1, M0  T1, N1, M0  T2, N0, M0 | Tumour <2cm and spread to 1 - 3 axillary lymph nodes, or Tumour < 2 cm and internal mammary LNs are positive for micrometastases on sentinel LN biopsy, or Tumour 2 - 5 cm, with no LN spread |
| **IIB** | T2, N1, M0  T3, N0, M0 | Tumour 2 - 5 cm, spread to 1 - 3 axillary LNs lymph nodes and/or internal mammary LNs are positive for micrometastases on sentinel LN biopsy Tumour > 5 cm, but does not grow into the chest wall or skin and has not spread to LNs |
| **IIIA** | T0-2, N2, M0  T3, N1-2, M0 | Tumour < 5 cm, spread to 4 - 9 axillary LNs, or enlargement of the internal mammary LNs Tumour > 5 cm across, has spread to 1 – 9 axillary LNs, but has not spread to the chest wall, skin, or internal mammary LNs |
| **IIIB** | T4, N0-2, M0 | The tumour has grown intro the chest wall or skin, and one of the following applies:  No LN spread  Spread to 1 - 3 axillary LNs lymph nodes and/or internal mammary LNs are positive for micrometastases on sentinel LN biopsy Spread to 4 - 9 axillary LNs, or enlargement of the internal mammary LNs |
| **IIIC** | T0-4, N3, M0 | The tumour is any size, and one of the following applies:  Cancer spread to ≥10 axillary LNs Spread to LNs under, and/or above the clavicle Involves axillary LNs, and with enlargement of the internal mammary LNs Involves ≥ 4 axillary LNs, and internal mammary LNs are positive for micrometastases on sentinel LN biopsy |
| **IV** | T0-4, N0-3, M1 | Tumour any size, but spread to distant organs or to LNs far from the breast. Includes inflammatory breast cancer |

**Supplemental Table 1 (on-line only).** Breast cancer staging by ‘TNM grouping’, based on the American Joint Committee on Cancer (AJCC) Tumour Nodes Metastases (TNM) system for staging breast cancer (6^th^ Edition; Greene *et al*, 2002).

| **Stage** | **0** | **I** | **II-i** | **II-ii** | **III** | **IV** | **Total** |
| --- | --- | --- | --- | --- | --- | --- | --- |
| **Total No patients** | 348 | 992 | 859 | 182 | 224 | 7 | 2612 |
| **Staging Investigations:** | | | | | | | |
| **CXR** | 136 | 527 | 580 | 148 | 160 | 5 | 1556 |
| **US** | 0 | 19 | 101 | 100 | 114 | 5 | 339 |
| **BS** | 0 | 22 | 109 | 113 | 125 | 4 | 373 |
| **CT** | 3 | 6 | 19 | 15 | 34 | 1 | 78 |
| **Total No Pts having staging investigations** | | | | | | | |
| **True +ve (%)** | 0 (0) | 0 (0) | 2 (0.3) | 10 (6) | 26(13.9) | 4 (57) |  |
| **False +ve (%)** | 3 (2.2) | 14 (2.6) | 23 (3.8) | 22 (13.1) | 24 (12.8) | 0 (0) |  |

**Table 2.** Number of investigations performed by modality and stage of disease and true positive and false positive results by stage of disease. Stage II-i = patients with stage II disease by AJCC 6^th^ edition (Greene *et al*, 2002): ≤ 3 lymph nodes positive, stage II-ii = patients with stage II disease by AJCC 5^th^ edition (Fleming *et al*, 1997), but stage III disease by AJCC 6^th^ edition, i.e. ≥ 4 positive lymph nodes.

|  | **CXR** | **US** | **BS** | **CT** |
| --- | --- | --- | --- | --- |
| **Total** | 1556 | 339 | 373 | 78 |
| **True +ve (%)** | 3 (0.2) | 6 (1.8) | 23 (6.2) | 21 (26.9) |
| **False +ve (%)** | 20 (1.3) | 13 (3.8) | 51 (13.7) | 3 (3.8) |

**Table 3.** True positive and false positive results by modality (includes all stages of disease).

|  | **TNM stage** | | | | | |
| --- | --- | --- | --- | --- | --- | --- |
|  | **0** | **I** | **II-i** | **II-ii** | **III** | **IV** |
| **True positive rate**  **for detection of metastases (%)** | 0 | 0 | 0.2 | 5.5 | 11.6 | 57.0 |
| **Estimated cost of detecting 1 patient with metastatic disease by:** | | | | | | |
| **“Standard” Staging Investigations:**  **(CXR,US,BS)** | Infinite | Infinite | £200,393 | £8,492 | £4,021 | £817 |
| **CT staging alone** | Infinite | Infinite | £119,744 | £5,074 | £2,405 | £488 |

**Table 4**

Estimate of healthcare costs for detect metastases by stage of disease and mode of imaging. Estimates for detecting a single patient with metastatic disease for each disease stage based on local costings and the respective true and false positive rates by modality (see text for costing estimates).

**Figure Legends**

**Figure 1.** An ‘unequivocal’ true positive. Chest X-ray showing multiple metastases throughout both lungs in a patient with stage IV breast cancer.

**Figure 2.** True positive. Chest X-ray reveals a ‘coin’ lesion in the left mid-zone, also demonstrated on the lateral radiograph (b). CT examination (c) on lung-window settings supports the diagnosis of a likely lung metastasis, later confirmed by CT-guided biopsy, performed with the patient lying prone (d).

**Figure 3.** True positive. Stage IV breast cancer patient. Ultrasound liver shows irregular lesions within the liver (a), subsequent CT (b) confirms liver metastases, ascites also demonstrated.

**Figure 4.** False positive. Bone scintigram (a) and CT chest examination (b, c, d) in a patient with stage IV breast cancer. The bone scan was reported as showing hot spots in the T10 vertebrae, left posterior 7^th^ and right anterior 2^nd^ ribs, consistent with metastases. The follow-up CT scan revealed these changes to be due to rib fractures (b), and degenerative disease (c, d) only.

**Figure 5.** One of two patients with stage II-i disease with a ‘true positive’ diagnosis. The soft tissue lesion in the right lower zone was reported as being a metastatic lesion on baseline CXR shortly after the patient presented with primary breast cancer in 2001 (a). However, in 2007 the lesion had not significantly changed in size and was retrospectively described as being ‘unlikely to be malignant’.
